# ID 88 - Eficácia, Segurança e Custo-Efetividade do Rastreamento para o Câncer Colorretal: Revisão Sistemática

**DOI:** 10.5327/2237-9622.2025.v34s1.88

**Published:** 2025-11-25

**Authors:** Rita de Cássia Ribeiro de Albuquerque, Cláudia Lima Vieira, Ana Paula Rodrigues Siqueira, Raphael Duarte Chança, Isabel Cristina de Almeida Santiago, Ricardo Ribeiro Alves Fernandes, Laura Augusta Barufaldi

**Keywords:** rastreamento, diretrizes, neoplasias colorretais, Avaliação de Tecnologias em Saúde

## Abstract

**Introdução:**

O câncer colorretal (CCR) é passível de ações de rastreamento. Contudo, não é isento de riscos e seus eventos adversos (EA) devem ser monitorados, assim como a custo-efetividade deve ser analisada. Foi realizada revisão sistemática (RS) para avaliar eficácia, segurança e custo-efetividade do rastreamento de CCR, definir sua relevância e eventual adoção no Brasil.

**Método:**

Entre novembro de 2023 e janeiro de 2024 foram conduzidas buscas no MEDLINE, Embase e Cochrane Library sem restrição para idioma. Para desfechos de eficácia e segurança foram recuperados 594 resultados, excluídas 84 duplicatas e avaliados 510 títulos e resumos. Cinco seguiram para a leitura de texto completo e apenas uma RS atendeu aos critérios de elegibilidade. Sobre avaliação de custo-efetividade (ACE) foram realizadas buscas por RS no MEDLINE, Embase e SCOPUS. Foram recuperados 501 registros e removidas 206 duplicatas. Após a leitura de títulos e resumos restaram 295 registros e desses 37 foram lidos na íntegra, sendo selecionadas duas RS.

**Resultados:**

Rastreamento com retossigmoidoscopia reduziu incidência (RR: 0,76; IC 95%: 0,70-0,83) e mortalidade (RR: 0,74; IC95%: 0,69-0,80) por CCR, quando comparado à ausência de rastreamento (nível de evidência alto). Rastreamento com exame guaiaco de sangue oculto nas fezes (g-FOBT) anual e bienal, comparado à ausência de rastreamento, reduziu mortalidade (anual = RR: 0,69 [IC 95%: 0,56-0,86]; bienal = RR: 0,88 [IC 95%: 0,82-0,93; nível de evidência moderado e alto, respectivamente). Na comparação entre rastreamento com g-FOBT anual versus g-FOBT bienal também houve redução na mortalidade (RR: 0,79 [IC 95% 0,64-0,98]; nível de evidência moderado). O perfil de segurança do rastreamento para CCR com as intervenções investigadas foi considerado adequado [nível de evidência moderado para retossigmoidoscopia e colonoscopia; nível de evidência baixo para g-FOBT e teste imunoquímico (FIT)]. As RS sobre ACE incluíram estudos conduzidos na Europa e EUA. A RS realizada por Diedrich e colaboradores selecionou 25 estudos que apresentaram a sigmoidoscopia como estratégia isolada ou combinada em comparação à ausência de rastreamento ou com outras estratégias já implementadas nos países dos estudos. A sigmoidoscopia, na maioria dos estudos, mostrou dominância ou razão de custo-efetividade incremental (RCEI) nas faixas até 20 mil dólares em comparação à ausência de rastreamento. Na RS de Ramos e colaboradores foram incluídos 79 estudos cujas intervenções eram exames endoscópicos ou com material fecal, combinados ou não, e desses 21% demonstraram ser dominantes em relação à ausência de rastreamento.

**Conclusão:**

Redução de incidência e mortalidade por CCR com retossigmoidoscopia; redução de mortalidade com testes de sangue oculto nas fezes e perfil de segurança adequado, para a eventual adoção do rastreamento de base populacional no Brasil. Com relação às ACE, foi identificada relação custo-efetiva entre qualquer uma das estratégias analisadas e a ausência de rastreamento. No entanto, esses estudos foram conduzidos em países desenvolvidos, com diferentes limiares de custo-efetividade e diversidades nos sistemas de saúde. A implementação de um programa de rastreamento de CCR requer avaliação do contexto do País, do impacto orçamentário, da capacidade operacional das instituições de saúde, da qualificação dos profissionais e da cadeia de ações logísticas necessárias.

